# Structured information during the ICU stay to reduce anxiety: study protocol of a multicenter randomized controlled trial

**DOI:** 10.1186/1745-6215-10-84

**Published:** 2009-09-14

**Authors:** Steffen Fleischer, Almuth Berg, Thomas R Neubert, Michael Koller, Johann Behrens, Ralf Becker, Annegret Horbach, Joachim Radke, Mathias Rothmund, Oliver Kuss

**Affiliations:** 1 Institute of Health and Nursing Science, Medical Faculty, Martin-Luther-University Halle-Wittenberg, Germany; 2 Department of Nursing Research, University Hospital Giessen and Marburg, Location Marburg, Germany; 3 Center for Clinical Trials, University Hospital Regensburg, Germany; 4 Sana Herzchirurgische Klinik Stuttgart, Germany; 5 Department 4: Health and Social Work, University of Applied Sciences, Frankfurt/Main, Germany; 6 Department of Anesthesiology and Intensive Care Medicine, University Hospital, Martin-Luther-University Halle-Wittenberg, Germany; 7 Institute for Theoretical Surgery/Department of Visceral, Thoracic and Vascular Surgery, University Hospital Giessen and Marburg, Location Marburg, Germany; 8 Institute of Medical Epidemiology, Biostatistics, and Informatics, Medical Faculty, Martin-Luther-University Halle-Wittenberg, Germany

## Abstract

**Background:**

ICU stay is often associated with negative experiences for the individual patient. Many patients are disabled and their communication is restricted during the ICU stay. Specific information on procedures, sensations and coping behavior are thought to reduce anxiety on the ICU. Until now information programs to reduce anxiety were mainly delivered preoperatively, completely neglecting informational needs of non-elective ICU patients.

**Methods:**

The trial is designed as a prospective multicenter randomized controlled trial in the cities of Marburg, Halle and Stuttgart. Elective and non-elective ICU patients will be included. The trial includes an intervention and a control group on the ICU. The control group receives a trivial conversation without any ICU-specific information. The intervention group receives an information program with specific procedural, sensory and coping information about their ICU stay. Both conversations take place in the ICU and are planned to take about 10 minutes.

**Discussion:**

In contrast to former trials on information programs on the ICU-stay our intervention will take place in the ICU itself. This approach will ensure to compensate for memory effects due to anesthesia or preoperative stress. Further the results will be applicable to non-elective ICU-patients.

**Trial Registration:**

ClinicalTrials NCT00764933

## Background

ICU stay for ventilated patients is associated with severe impairments and limitations of communication. Additionally, these patients are restricted regarding their activities and ability to participate. These restrictions are mainly due to structural and procedural necessities including therapeutic interventions. The patients are not only handicapped by their vocal restraints, but also by the unknown and unfamiliar situation in the ICU. Nonetheless, ventilated patients are able to actively communicate with their environment. Research shows that nurse-initiated nurse-patient communication is lacking in this phase of ventilation [1]. If the nurses are interacting with the patient, the interaction is nonverbal and rather instrumental. With the patient getting more and more awake and able to respond, nurse-initiated communication increases [1].

Research into the communication behavior in intensive care units, not limited to ventilated patients, showed interactions and interaction conditions which had predominantly been perceived as problematic by patients [2-10]. ICU patients often experience these ineffective communication situations with feelings of anxiety, insecurity but also irritation. Particularly ventilated patients evaluate the communication during their ventilation as non-successful [3,10,11]. In this context, Alasad and Ahmad [2] found that communication with patients in ICUs is not continuous and strongly depends on the patient's condition. Intensive care nurses found work with awakened patients being able to communicate verbally as more exhausting, as compared to still unconscious patients. Here, communication was also not perceived as a possibility of informing and supporting the patient.

Similarly, Scheer [12] observed that the nursing staff in ICUs often feels overstrained and tends to withdraw from the patients' bedsides. Similar results were obtained by means of a video analysis which investigated the communication and interaction between ventilated patients and nursing staff in an ICU [1]. This analysis focused on the patient's remaining possibilities of expression in the different treatment stages taking into account also aspects of initiative and responsive communication. As a result, lessons in specific communicative abilities for nursing staff in intensive care were proposed, as the staff had acted upon "normal" social communication patterns and had not sufficiently observed the particularities of the setting [1].

The high priority of successful communication with communicatively challenged patients, however, is also appreciated by the ICU nursing staff [11,13]. In their qualitative study, Usher and Monkley [13] investigated the point of view of nursing staff on successful and effective communication in an ICU and were able to identify three main issues: "perception of caregivers", "presence" and "strain relief" - whereas the category "strain relief" is mainly made up of the situational information of the patient. This perception of successful conscious communication is opposed by the notion that communication is always something random and natural [13]. Against the background and as a consequence of Liedtkes [1] results, a concrete and conscious communication intervention for the information of the ICU patient and with "strain relief" in mind would be necessary.

Only one randomized controlled study (RCT), could be found which deals with a information intervention in the ICU [14]. For their research approach in cardiac surgery patients, Hwang et al. [14] chose a recorded tape information from the attending physician which was played to the patients after recovering from anesthesia. The contents of the 6 to 8 minute recordings included the course as well as the result of the operation and further treatment plans. The intervention group showed significantly superior values regarding anxiety, tension, depression and pain perception compared to the control group. The results of this study suggest the effectiveness of postoperative situational information intervention for a positive influence on psychological parameters. Therefore a nursing care information program comparable to and modeled on the brief medical information is to be tested in the planned trial with ICU patients.

### Trial objective

The aim of the project is to evaluate the effectiveness of a structured information program for patients who are staying in an ICU and thus having an associated induced communication restriction. In line with notions of control theory and nursing theory, the communication of knowledge is regarded as a means to enhance patients' capacity to understand, classify and predict the many unfamiliar events related to the ICU stay [15-17]. In this way it is not only possible to achieve an improvement of the difficult communication situation, but a cognitive reframing of the anxiety provoking ICU-stay. This can result in an increased perceived locus of control and eventually in stress reduction.

The key study hypothesis is:

An information program in the initial stage of treatment in an ICU alleviates unpleasant experiences related to the ICU stay and thus contributes to an improvement of the patient's situation and of particular aspects of quality of life, i.e. in relieving anxiety.

Besides this main point there are also other, secondary hypotheses particularly assuming effects on individual quality of life 3 months after discharge.

## Methods

The trial is designed as a multicenter trial with concealed random allocation and central data monitoring (coordinating center: Marburg; 3 study centers). The multicenter design was chosen to increase the generalizability of the study results. Within the framework of the Medical Research Council [18,19] this corresponds to the main phase of the evaluation of complex interventions, because the trial is designed as an extension to a previous study of our working group [20,21]. Study methods include the randomization on the patient level and the delivery of the intervention by members of the study group. Potential contaminations of the intervention and control group will be avoided by restricting recruitment to one patient per room on the ICU at the same time.

### Inclusion and exclusion criteria

Cardiac surgery, general surgery and internist patients with scheduled and unscheduled ICU stay (including High Dependency Units) will be eligible for inclusion in this trial. Exclusion criteria, besides a refusal to take part in the trial, are: no opportunity to fill in the mailed follow-up questionnaire, impaired cognitive ability and/or ability of judgment, language problems, accommodation in one room with another study patient, or age under 18 years.

### Recruitment of participants

**Figure 1:**
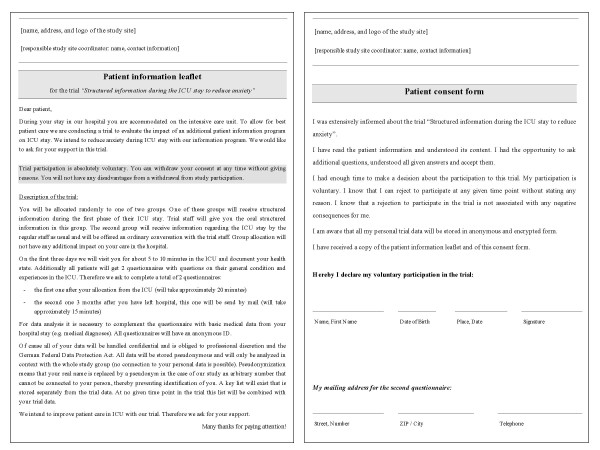
**Obtaining patient consent**. Patient information leaflet and patient consent form (translated version).

Information and instruction of the patients is done personally and in written form by executive persons, the consent is obtained in writing (see Figure 1).

All patients who are to be admitted electively to the ICU will be informed about the possibility of taking part in the trial before the transfer to the ICU or before the surgery and their informed consent will be obtained. This applies to the major part of cardiac surgery patients as well as to a minor part of the general surgery patients. All other patients, particularly those of internal medicine and trauma surgery, will be informed about the trial in the early stages of their intensive care stay and will be asked to take part. In case custodians are appointed, they will be informed about the possibility of taking part in the trial and will be asked for a participation of their charge in the trial.

### Randomization

The group allocation is done via concealed randomization (concealed allocation) on the patient level, stratified by center and unit into 2 study groups:

▪ structured information at the beginning of the ICU stay (*Intervention group*) or

▪ sham comparator, unspecific conversation (*Control group*).

The randomization list will be computer-generated before the start of the trial (Institute of Medical Epidemiology, Biostatistics, and Informatics, Halle/Saale). Concealed randomization will be realized by using opaque, consecutively numbered envelopes after the inclusion of the study patients.

### Blinding

Patients and field researchers are not blinded to group allocation. A blinded statistician not involved in the trial process will be responsible for the final analysis.

### Study procedure

The experimental intervention is planned as a conversation with ICU-specific information on the ICU during the first stage of the ICU stay. The control intervention is planned as a non-specific conversation of the same duration and at the same point of time as the experimental intervention.

During the information intervention a standardized and an individualized part are combined: a guided conversation corresponding to the individual choice in the dialogue. Thereby patients will be given sensory and procedural information (e.g. sources of noise in the ICU, chronology) as well as coping information (e.g.: What can you do if you are feeling strained?).

The first, standardized part of the intervention comprises information on nine topics, that have been identified in preliminary studies [22,23] as relevant for patients in ICUs. The topics of the standardized part of the information program are described in Table 1.

**Table 1:** Information intervention.

**Nr.**	**Topic**	**Details**
**1**	**People in the ICU**	• Health care professionals (nurses and intensive care nurses) • Attending physician • Clothing incl. specifics such as masks, gloves etc. • Change of shifts • Ward rounds

**2**	**Devices and monitoring**	• Monitor incl. central monitoring • Ventilator • Infusion and syringe pump (infusomat and perfusor) • Alarms

**3**	**Room furnishing**	• Clock • Bell system • Room size

**4**	**Individual safety**	• Tubes, drainages, wounds, urinary catheters, fixation • Tube, respiratory mask • Waking phase • Intravenous access • Bedding • Dimming of the light

**5**	**Schedule**	• Hospital stay duration • Transfer to IMC • Differences between IMC and ICU • Nutrition

**6**	**Communication**	• Nod, shake of the head • Pens

**7**	**Staff duties**	• Aspiration • Mobilization • Radiologic examinations • Personal hygiene/oral hygiene

**8**	**Conveniences**	• Analgesics and soporifics (pain relievers and sleeping pills) • Visiting hours • Information before nursing-medical interventions

**9**	**Helpful thoughts**	• Everything is done for me. That is sign that everything worked alright. • I don't have to suffer any pain; if necessary I will receive additional medication. In the meantime I can relax and continue to breathe calmly. • Only a little longer, then I have made it.

Topics of the information program.

In the second part of the intervention the patients' individual information need on particular topics will be determined and further addressed. In addition, the patient will be given the opportunity to receive information on other topics.

Following preliminary studies, a length of about 10 minutes is scheduled for the intervention [14]. A second intervention time will not explicitly be ruled out if the patient's information need could not be met with due to exhaustion, failing concentration or an interruption for other reasons.

The control patients will be offered a semi-structured, non-specific conversation with a member of the study group. This conversation with the patient will also take about 10 minutes. Priority will be given to topics such as mental state, experienced pain, sleeping habits and experienced anxiety.

Accompanying close relatives or friends can stay during the (control-) intervention if the participant agrees. As this can enhance the message, this will be documented in the case report form.

Before the experimental and control intervention the patients' level of consciousness and concentration will be recorded using the Richmond Agitation and Sedation Scale - RASS [24] and the Confusion Assessment Method for the ICU - CAM-ICU [25,26]. The interventions will only be carried out at RASS values ≥ -3 and a negative test outcome of the four CAM-ICU criteria. In case of lower values the patient will be re-assessed at a later date (→ 24 h interval).

The study procedure will be carried out in each center by one of the study members who are not involved in the standard care in order to receive information on the efficacy. The comparability of the intervention taking place over the different centers is guaranteed by preliminary instructions of the study personnel for the implementation of the information program and the control intervention. Additionally, the coordinating center in Marburg will centrally monitor the whole trial.

### Measures

#### Primary Outcome

**Figure 2:**
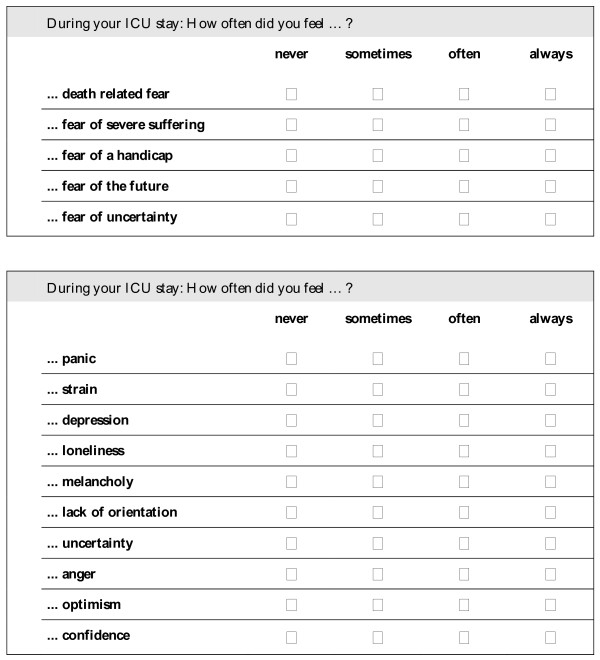
**Measurement of anxieties during the ICU stay**. Anxiety-related part of the CINT questionnaire (translated version).

The primary endpoint is the anxiety-related part of the CINT questionnaire on the experiences and the emotional state in the ICU which is being recorded at the time of admission to the standard ward. This questionnaire has already been used in preliminary studies [20,22] and represents specific aspects of the quality of life in relation to the ICU. The anxiety-related part corresponds to the experienced anxieties during the ICU stay and comprehends the following items: death related fear, fear of severe suffering, fear of a handicap, fear of the future, fear of uncertainty, panic, strain, depression, loneliness, melancholy, lack of orientation, uncertainty, anger optimism and confidence. All items are rated on a 4-point Likert-scale from "never" to "always" (see Figure 2).

#### Secondary Outcomes

In addition to the anxiety-related part of the CINT questionnaire a Visual Analogue Scale-Anxiety - VAS-A [27] supported by the Faces Anxiety Scale [28] is used. Evaluation of potential discrepancies between the retrospectively reported anxiety and the measured anxiety during the ICU-stay is of particular interest. The State and Trait Anxiety Inventory - STAI [29] as a further anxiety-related instrument will be recorded to compare anxiety after ICU-stay.

Furthermore the CINT questionnaire [20,22] measures additional parameters of the ICU related experiences.

As a secondary variable, the patients' level of consciousness and concentration regarding a potential ICU-caused state of confusion will be surveyed with the Confusion Assessment Method for the ICU - CAM-ICU [25,26]. The test procedure CAM-ICU, also appropriate for ventilated patients, comprehends cognitive as well as process-related components. These components are illustrated using the Richmond Agitation and Sedation Scale - RASS [24] and the four delirium main criteria "acute onset", "inattention", "disorganized thinking" and "altered level of consciousness".

For the analysis of additional effects the duration of stays (ICU, intermediate care, standard ward, hospital) and routine date on the course of inpatient treatment are recorded. Socio-demographic data like age, gender, marital status and routine treatment data like medical diagnoses will be documented thoroughly.

Uncertainty tolerance will be measured using the Uncertainty Tolerance Scale [30] and semi-structured questions on convalescence are asked to get a qualitative impression of the participants health state.

Expected results will contribute to an improved communication in the ICU and finally to an improved quality of life as a desired treatment goal [31-33]. Therefore we will measure quality of life using the Schedule for Evaluation of Individual Quality of Life - SEIQoL [34], and the SF-12 [35]. For this reason we implemented a paper questionnaire version of the SEIQoL to test for acceptance of a self evaluation form for this kind of quality of life questionnaire.

All measurements and the timeline are summarized in Table 2.

**Table 2:** Measurements.

t_0_	day of recruitment (ICU)	▪ Socio-demographic data, routine treatment data^2^ ▪ Acute ICU-Syndrome (CAM-ICU incl. RASS)^2^ ▪ Anxiety (VAS-A)^1^
t_1_	24 h after study intervention	▪ Acute ICU-Syndrome (CAM-ICU incl. RASS)^2^ ▪ Anxiety (VAS-A)^1^

t_2_	48 h after study intervention	▪ Acute ICU-Syndrome (CAM-ICU incl. RASS)^2^ ▪ Anxiety (VAS-A)^1^

t_3_	admission to standard ward	▪ Experience and emotional state in the ICU (CINT-FB)^1^ ▪ Anxiety (VAS-A)^1^ ▪ State-Anxiety (STAI-State)^1^ Estimated time for the questionnaire: 20 minutes

t_4_	discharge from hospital	▪ In-patient history and postoperative complications^2^ ▪ Length of stay and mode of discharge^2^

t_5_	3 months after discharge (by mail)	▪ Individual quality of life (SEIQoL)^1^ ▪ Quality of life (SF - 12)^1^ ▪ Uncertainty (Uncertainty Tolerance Scale)^1^ ▪ Convalescence (Semi-structured questions)^1^ ▪ Trait-Anxiety (STAI-Trait)^1^ Estimated time for the questionnaire: 15 minutes

Points of measurement and outcome measures according to case report form.

^1^Self evaluation; ^2 ^Third-party evaluation

### Sample size calculation

The sample size calculation is based on the anxiety-related part of the CINT questionnaire as particular aspects of the ICU-patients' quality of life [21]. This sum score can be represented on a scale from 0 to 100 and has added up to a mean value of M = 28.0 and a standard deviation of SD = 17.0 in an earlier unpublished trial. The basis for the sample size calculation was formed by the following assumptions: α = 0.05; β = 0.20; Δ = 8.50 scale points. The determination of the expected difference Δ was done on the notion that in quality of life measures the differences within the scope of half a standard deviation are considered as minimally clinically relevant [36]. Under these conditions at least 70 patients per group have to be included to ensure the effect statistically. In order to compensate potential drop-outs the sample size was determined n = 100 per group, corresponding to a total sample size of N = 200 (n = 100 vs. n = 100). In the centers Stuttgart and Halle respectively n = 30 patients per study group will be recruited, in Marburg n = 40 per study group.

### Drop-outs

Drop-outs will be documented thoroughly and included in the data analysis until the point of drop-out. Reasons for drop-out will be reported and analyzed.

### Data analysis

Descriptive analyses will be carried out by standard methods, taking into consideration the scale of the respective variables. For analysis of the primary endpoint we will use an ANCOVA model with the primary endpoint as the response, and the center as a covariate, thus allowing for the stratified randomization procedure. The treatment effect will be reported as an adjusted difference in means with a corresponding 95% confidence interval. Secondary endpoints will be assessed with the respective adjusted models, using standard ANCOVA models for continuous outcomes and logistic regression for binary outcomes. In case of repeatedly measured outcomes, mixed models will be used for analyses to adjust for within-patient correlations [37]. Secondary endpoints analyses are considered as merely exploratory. The analysis of all outcomes will obey to the intention-to-treat principle. Subgroup analyses will be conducted as interaction tests, also in a non-confirmatory fashion [38].

### Protection of data privacy

We will create a pseudonym for all trial participants to collect and analyze the trial data. Key lists will be stored separately from the trial data and erased after final data analysis. Data will be analyzed in a way that no conclusions can be drawn to individual participants. Trial data is stored in lockable cabinets in lockable rooms.

### Quality assessment

The trial is part of the Nursing Research Network "Mitte-Süd". A report system is established within the network. Annual quality reports have to be prepared for the German Federal Ministry of Education and Research.

As the process of a multicenter trial demands high standards of quality to warrant comparable conditions and results among the centers all procedures were developed and documented in joint commissions.

### Publication policy

We plan to publish the trial results in a peer-reviewed, international, Medline-listed journal, independent of study results. This mainly serves the purpose to avoid publication bias. Additionally, we are obliged by the Federal Ministry of Education and Research to report our results within 6 months after study termination. All trial results will be reported within context to this study protocol.

### Ethical considerations

The study protocol is approved by the ethics committees of the universities in Marburg, Halle and Tübingen. If changes to the study procedures are necessary they will be proposed to the local ethics committees as amendments. All changes will be described and discussed in the publication of the trial's results.

## Discussion

In contrast to former trials on information programs on the ICU-stay our intervention will take place in the ICU. This approach will ensure to compensate for memory effects due to anesthesia or preoperative stress. Further the results will be applicable to non-elective ICU-patients. Thus our research will particularly contribute to the evidence on ICU-related information and communication.

## Competing interests

The authors declare that they have no competing interests.

## Authors' contributions

SF, AB, TRN, MK, JB and AH were responsible for the general study design. OK and MK planned the statistical analysis, carried out the sample size calculation and were responsible as biometric counselors. TRN, JB, JR, MR and AH are the responsible project coordinators in the three participating study centers and the main investigators. RB is responsible for central monitoring. All authors were responsible for the drafting of this paper and approved the final manuscript.
